# What do users think about Virtual Reality relaxation applications? A mixed methods study of online user reviews using natural language processing

**DOI:** 10.1016/j.invent.2021.100370

**Published:** 2021-02-02

**Authors:** Simon Fagernäs, William Hamilton, Nicolas Espinoza, Alexander Miloff, Per Carlbring, Philip Lindner

**Affiliations:** a Centre for Psychiatry Research, Department of Clinical Neuroscience, Karolinska Institutet, & Stockholm Health Care Services, Stockholm, Sweden; b Mimerse, Stockholm, Sweden; c Gavagai, Stockholm, Sweden; d Department of Psychology, Stockholm University, Stockholm, Sweden

**Keywords:** Virtual reality, Mental health, Natural language processing, User experience, Relaxation

## Abstract

The advent of affordable Virtual Reality (VR) technology has spurred consumer and commercial interest in VR relaxation applications, which has quickly grown into a popular non-gaming genre on digital marketplaces. While laboratory studies have demonstrated efficacy of VR relaxation for mental health purposes, little is known about how users experience this type of intervention and no study has examined the reception of consumer versions among regular users in everyday life. Studying published user reviews offers a unique window into naturalistic user experiences that complements traditional qualitative methods by circumventing the sampling bias of interview studies, and allowing analyses on full samples, unconstrained by coding resources. Using an innovative, semi-automated Natural Language Processing technique, the current study analyzed 1379 published reviews (including star ratings) of 30 different VR relaxation applications available for the Oculus Go and Gear VR. The uncovered topic structure and sentiment analysis thereof suggests that users have an overall positive view of VR relaxation applications, describing them as successful in inducing immersion and relaxation, and having appreciated gamification elements. However, perceived quality varied substantially between applications that explained more variance in star ratings than specific features. Critical issues raised were both technical (e.g. “overheating”) in nature and related to specific design elements and use. Implications for the design of consumer VR applications and future research are discussed.

## Highlights

• Guided, natural language processing generated a valid topic model of content expressed in user reviews
• Consumer Virtual Reality relaxation applications are viewed positively by users
• Perceived quality varied extensively between applications

## 1 Introduction

Virtual Reality (VR) is an immersive technology that creates the experience of being present in a virtual environment, today typically achieved using a head-mounted display that withholds the outside world, measures head rotation continuously and adapts the stereoscopic view accordingly (Scarfe and Glennerster, 2019). In addition to applications like games, immersive storytelling and other popular forms of entertainment, these virtual environments can be designed for clinical purposes by translating therapeutic mechanisms into a VR experience (Lindner, 2020). In its simplest form, this involves a virtual simulation of an environment or action otherwise performed for the same purpose in real life. Although therapeutically simple, such virtual scenarios may nonetheless carry substantial practical benefits: VR exposure therapy for phobias for example (Botella et al., 2017), solves many of the logistic issues in providing exposure therapy by enabling exposure to otherwise unavailable stimuli (Lindner et al., 2017, Lindner et al., 2020a), allows tailoring of stimuli (Rizzo et al., 2010) and makes it fully controllable. VR exposure therapy has been shown to be efficacious by numerous meta-analyses (Carl et al., 2018; Fodor et al., 2018; Wechsler et al., 2019) and new research has demonstrated efficacy also in an automated, gamified format (Donker et al., 2019, Donker et al., 2020; Freeman et al., 2018; Lindner et al., 2020b; Miloff et al., 2019) that makes more extensive use of the capabilities inherent to the technology, e.g. by featuring an embodied virtual therapist (Miloff et al., 2020).

Just as VR technology can be used to induce a fear response by presenting virtual equivalents of phobic stimuli in an immersive manner (enabling exposure therapy), it can also be used to induce relaxation by situating the user in a peaceful virtual environment, often depicting nature settings like beaches and forests. Voice-over instructions for breathing and meditation exercises, or simple and repetitive motor or cognitive tasks, can be added to provide a more active form of relaxation. A multitude of studies relying on both subjective and objective measures of relaxation have shown that this approach does indeed induce relaxation and/or lowers stress (Anderson et al., 2017; Annerstedt et al., 2013; Hedblom et al., 2019; Serrano et al., 2016; Valtchanov et al., 2010). The recent advent of affordable consumer VR technology, including mature digital ecosystems for application distribution, presents a paradigm shift in the availability and scalability of VR mental health, including VR relaxation. One such first-generation VR application released in 2016, saw over 40,000 users over two years, despite a very limited VR user base at the time, revealing a promising public health potential (Lindner et al., 2019). Since then, VR relaxation has grown into one of the most popular non-entertainment categories on application marketplaces. The high population prevalence of stress (Bergdahl and Bergdahl, 2002; Nordin and Nordin, 2013) and other mental health problems that can be alleviated by relaxation (Klainin-Yobas et al., 2015), in combination with relatively uncomplicated software development – in its simplest form requiring only a set of high-definition 360° nature videos, soothing music and a basic user interface – has manifestly provoked a large commercial interest in the genre and there are now a multitude of applications available at digital marketplaces.

Early efficacy research on VR relaxation aside, we are not aware of any research showing that this efficacy translates into real-world effectiveness when used by ordinary users at home, outside research contexts. Seemingly great variations in content and quality across applications also make it unlikely that all published applications share the same effectiveness and user engagement. Related research on smartphone applications for depression (Larsen et al., 2019; Shen et al., 2015) suggests that few commercial VR relaxation applications can be expected to be evidence-based and thereby effective. Uptake and usage statistics from the first-generation VR relaxation application mentioned above suggested a low degree of recurrent users, although lack of individual data precluded definitive conclusions (Lindner et al., 2019). Further, the extant qualitative literature on user experiences of VR relaxation is very limited, in particular research using applications of the type that is now commercially popular. A recent study identified nine themes from interviews with users who tried a VR mindfulness paradigm that included 360° video of a tranquil forest: the themes that emerged related to mindfulness practices as well as more generic VR aspects like variable and interrupted sense of presence, the need for personalization, the importance of graphical quality and the head-mounted display (Seabrook et al., 2020). The fact that the relaxation was carried out in a laboratory setting, that the VR paradigm was explicitly mindfulness-focused and included passive (i.e. non-interactive) 360° video rather than a computer-generated environment, does however raise important questions of how themes and implications generalize to other settings (i.e. at-home use) and to other types of relaxing environments and paradigms.

The current study employs a novel approach in examining experiences of using VR relaxation applications outside of a research context, in order to inform the development of the next generation of such applications. By mining and analyzing publicly available online user reviews of VR relaxation applications for two of the most popular consumer VR devices, the current study complements traditional, in-depth qualitative research by surveying a considerably larger sample of users (with presumably lesser sampling bias) who have used the applications under real-world conditions, offering maximum external validity of findings and excellent conditions to uncover a greater thematic width. Analysis of user reviews has previously been used in research on smartphone applications for depression (Stawarz et al., 2018), sleep problems (Aji et al., 2019), weight loss (Frie et al., 2017) and medication adherence (Park et al., 2019), as well as VR exergames (Faric et al., 2019). Unlike previous research that has typically relied on subjective manual coding, we used a semi-automated Natural Language Processing (NLP) tool (Espinoza et al., 2018) to uncover a topic structure from free-text data. This allowed us to analyze the full sample of available reviews (without random subsampling due to limited coding resources) using a replicable analysis still grounded in subject matter expertise. This analysis also enabled synergistic quantitative analyzes through topic modeling on a review level, allowing us to calculate topic prevalence across applications and application types (e.g. active versus passive relaxation), as well as calculating associations with accompanying star ratings. Based on analogous findings in the broader extant mHealth literature, we hypothesized that user reception would vary greatly between applications, expressed both in terms of star ratings and thematic structure.

## 2 Methods

### 2.1 Ethics

The current study exclusively uses data that has manifestly been made public by each legal subject. The data is freely available online, the content of which is not considered sensitive according to the GDPR article 9 definition, referred to in Swedish legislation. This research thus falls outside the applicability of the Swedish Act Concerning Ethical Review of Research Involving Humans (2003:460), and no independent ethical review is therefore required. The Swedish Ethical Review Authority confirmed this interpretation (2020-06678). Identifying attributes were removed at the earliest possible stage. Due to reasons of integrity and methodological congruence, terms (the level of data used for analysis) rather than full quotes are presented in the Results.

### 2.2 Data collection and pre-processing

A number of inclusion criteria applied for data collection. This study focuses exclusively on applications for two mobile VR devices without position tracking (so called 3 degrees of freedom) (Scarfe and Glennerster, 2019): the Oculus Go and Samsung Gear VR. The Oculus Go is binary compatible with the Gear VR, and most applications can be launched without modifications from both devices. For this reason, although the official Oculus marketplace has separate Go and Gear VR sections, reviews of applications available on both devices are merged and it is not technically possible to disentangle which device was used by a particular reviewer (unless explicitly mentioned in text). We chose to limit our review mining to applications for the Go and Gear VR for several reasons. Since topic structure is modeled on a corpus level, including reviews that used newer devices with positional tracking – and in the case of the tethered Oculus Rift, also featuring greater graphical capabilities – was deemed likely to introduce unnecessary variation in relation to study aims. In addition, the Go and Gear VR are among the most popular VR devices and have been available for many years (allowing a large number of reviews to accumulate), with no or little relevant change in hardware in the last few years (respectively) that could introduce confounding variations in review content. Importantly, both these mobile devices are affordably priced and have had stable pricing over time (the Go costing approximately 240 USD), thereby introducing the least sampling bias in who can afford to acquire one. Finally, these devices have multiple popular relaxation applications available for download in a distinct and specified application category at the official marketplace, including free-text reviews in English with an accompanying numeric rating in the form of 1—5 stars (lowest to highest) for each listed application.

To collect reviews for analysis, a custom Python script was developed that mined all published reviews (including star ratings and current price) of all applications belonging to the “Relaxation and meditation” category on the official marketplace for the Oculus Go (an inclusion criteria). Only the Go marketplace was mined since this is the newer device of the two, with most applications offering backwards compatibility with the Gear VR (potential applications available for the Gear VR but not Go were deemed too old to be relevant). The script was run on 2020-03-23, resulting in an initial *n* = 1661 reviews of k = 56 applications.

**Fig. 1:**
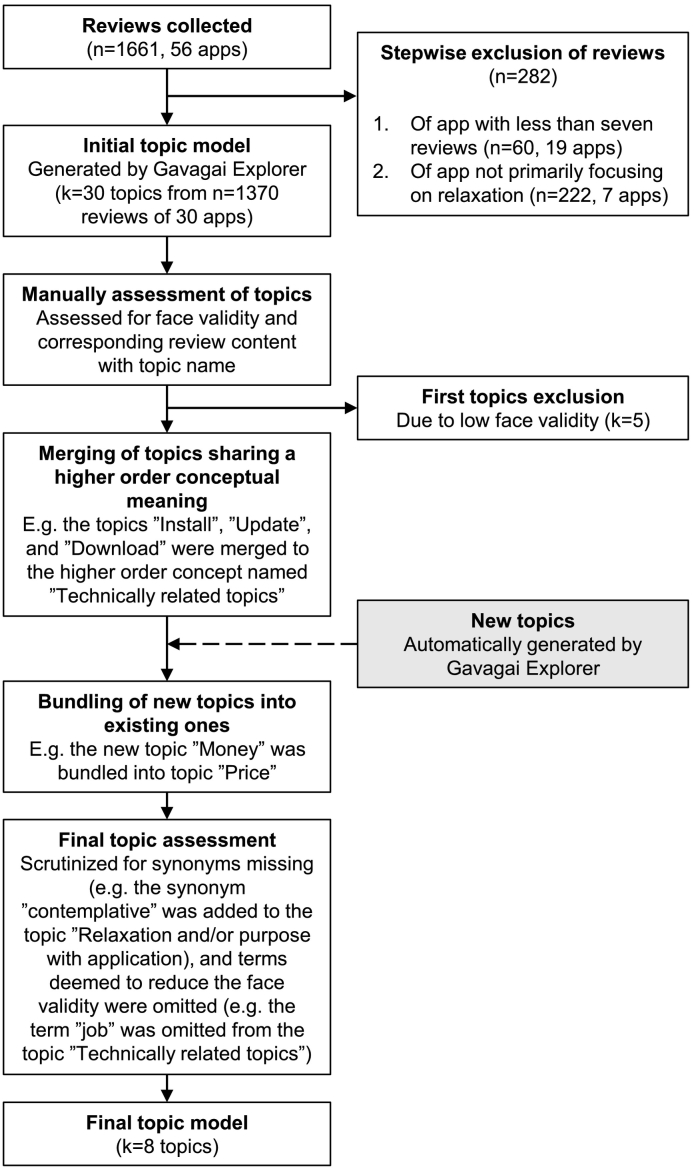
Analysis flowchart.

**Fig. 2:**
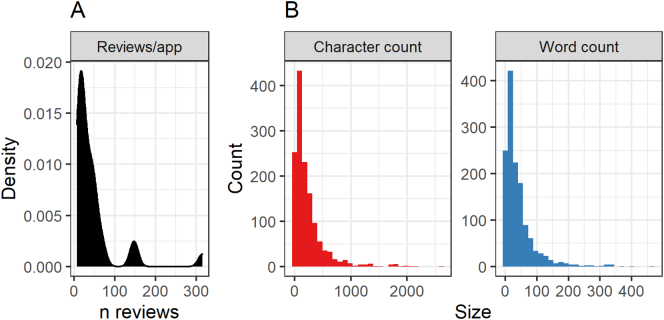
Distribution of number of reviews per application, along with character and word counts of reviews Panel A: Density plot of number of reviews per application. Panel B: Histograms of character (left) and word (right) counts of reviews.

Of these, *n* = 60 reviews belonging to k = 19 applications were then discarded due to having less than seven published reviews: in lieu of any established, applicable guidelines for this type of analysis, the choice of seven was guided by findings on the typical smallest sample size observed in qualitative health research (Vasileiou et al., 2018). The remaining applications were reviewed manually, resulting in k = 7 applications (with a total of *n* = 222 reviews) being excluded for not being primarily a relaxation application despite belonging to the relaxation category. Most of these excluded applications were either non-specific experience application (not marketed as a relaxation tool or any mention of such a component in the description) and/or primarily a puzzle game. The final, pre-processed dataset included k = 30 applications with a total of *n* = 1379 reviews posted by k = 1214 users, with a range of 7—316 reviews per application and a median of 23.5 reviews. See Fig. 1 for flowchart. All extracted and retained reviews were in English. See panel A of Fig. 2 for density plot of number of reviews per application. Median number of characters and words per review were 138 and 25, respectively; see panel B of Fig. 2 for histograms.

To allow analyses on not just individual applications but application features, the final k = 30 applications were classified by author AM using a novel coding scheme developed specifically for this study, in lieu of any consensus classification system in the extant literature for VR relaxation applications. This classification scheme included four variables: type (meditation or other, e.g. game, sport or art), engagement (primarily passive or active), environment style (natural or unnatural, e.g. psychedelic), and price (collapsed into either free or not free). Two additional categorizations were initially considered but dropped during course of categorization: sandbox features and primary modality (audio or video). The former categorization was dropped due to difficulties in thresholding the extent of customization options, while the latter was dropped due to most applications emphasizing both modalities. See Table 1 for description of included applications.

**Table 1:** Classification of applications included.

Name	Also available on gear VR	Type	Engagement	Environments	Price^a^
Big Breezy Boat	Yes	Other	Active	Natural	Not free
Binaural Waves Meditation	Yes	Meditation	Passive	Both	Not free
Calm	Yes	Meditation	Passive	Natural	Free
Calm Place	Yes	Meditation	Passive	Natural	Free
Chakra VR	Yes	Meditation	Passive	Unnatural	Not free
Chimera Reader	No	Other	Active	Natural	Free
Claro	No	Other	Active	Unnatural	Not free
Cosmic Flow: A Relaxing VR Experience	No	Meditation	Passive	Unnatural	Free
Deepak Chopra Finding Your True Self	Yes	Meditation	Passive	Unnatural	Not free
exVRience relaxation	Yes	Meditation	Passive	Natural	Not free
FlowVR Meditation	Yes	Meditation	Passive	Natural	Not free
Forest of Serenity	Yes	Meditation	Passive	Natural	Free
GoPaint	Yes	Other	Active	Both	Not free
Guided Meditation VR	Yes	Meditation	Passive	Natural	Free
Guided Relaxation VR	No	Meditation	Passive	Natural	Free
Happy place	Yes	Meditation	Passive	Natural	Free
Healium	Yes	Meditation	Passive	Both	Free
Jigsaw 360	No	Other	Active	Both	Not free
Liminal	No	Multiple	Active	Both	Free
Meditainment VR	Yes	Meditation	Passive	Both	Free
Nature Treks VR	Yes	Meditation	Active	Natural	Not free
Paddle Ride Experience	Yes	Other	Active	Natural	Not free
Real VR Fishing	No	Other	Active	Natural	Not free
Relax with Nature VR	Yes	Meditation	Passive	Natural	Not free
ShapeSpaceVR - Zen Parade	Yes	Meditation	Passive	Unnatural	Not free
Sphaeres	Unknown	Meditation	Passive	Natural	Not free
TRIPP	No	Multiple	Both	Both	Free
WORLDS	No	Multiple	Passive	Natural	Not free
VR Church: The Bible	Yes	Multiple	Active	Natural	Free
Zen Zone	Yes	Meditation	Both	Unnatural	Not free

a At time of data mining. Prize is for application download – additional costs for content may apply.

Before exporting the pre-processed data for NLP, application and user names were blinded by replacement with unique, random alphanumerical sequences. To preserve scientific neutrality, and since the goal of the current study was to investigate user experience of the relaxation-type of applications rather than evaluate specific applications, only blinded application names are presented in the results.

### 2.3 Topic modeling through semi-automated natural language processing

The blinded, preprocessed data set was uploaded to the Gavagai Explorer web interface. In contrast with other NLP approaches, this tool was explicitly designed for semi-automated analysis using a stepwise, incremental process of refining clustering structure and correcting errors (Espinoza et al., 2018). In brief, Gavagai Explorer performs lexical clustering by estimating relative neighborhood topology in semantic space (Gyllensten and Sahlgren, 2015), at each iteration adding or suggesting synonyms to increase topic coverage based on an online distributional semantic model available in multiple languages (Sahlgren et al., 2016). On the assumption that choice of wording reflect underlying emotions, and that words thus carry attitudinal loadings, this method also allows estimation of sentiments (attitudes towards something, driven by emotions) through lexical counting and aggregation (Karlgren et al., 2012). In the current study, individual reviews were classified using the common positive-negative polarity (Fang and Zhan, 2015).

The analysis pipeline began by automatically generating an initial model set to show the 30 top-ranked topics, with topic ranking based on occurrence in the data. The initial model was then refined iteratively and stepwise by author SF to create a meaningful, comprehensive and condensed topic structure, in a process similar to that used in thematic analysis (Braun and Clarke, 2006). In a first step, the researcher read several reviews assigned to each preliminary topic to become familiar with the topic and to assess face validity based on the correspondence between topic name and the content expressed. The face validity was assessed manually on a scale from one (low face validity) to five (high face validity). In the second step, topics with ratings of one were omitted from the model. In step three, to reduce the number of topics, topics sharing a higher order concept were merged and renamed to capture the higher order concept. This process was then repeated in step four. When two topics are merged, the Gavagai Explorer tool automatically generates a new topic placed last in the ranking list. If the new topic was assessed to share conceptual content with an already existing topic, it was merged into it, otherwise it was saved as a new topic. The topic structure was deemed saturated when the automatically generated topics was not assessed as a new topic for at least 5 times in a row. Finally, the included terms belonging to each topic were scrutinized. Terms deemed to reduce the face validity of the topic was omitted. Synonymous, misspelt and/or complementary terms that were missing were added, aided by the tool's automated suggestions, although at this stage, this had very minor impact on the final topic structure and sentiments.

### 2.4 Statistical analyses

All statistical analyses were conducted in the R 3.6.3 environment. Differences in occurrence of each individual topic and overall review sentiment (respectively) across application were examined using chi-square tests, while differences across applications in star rating was examined using Welch's F test. Separate logistic regression models (predicting overall review sentiment) or linear regression models (predicting star rating) were used to examine the impact of each specific topic while controlling for occurrence of the others. Finally, the impact of application feature categories was examined by running a linear regression models featuring the four categorizations as predictors of star rating. For this analysis, we omitted k = 229 reviews from ten applications which could not be validly categorized in a binary manner, e.g. due to featuring both natural and unnatural virtual environments. This model was then re-run as a random-intercept mixed effects model (Bates et al., 2015; Kuznetsova et al., 2017) in order to examine whether any differences across feature categories remained after taking into account clustering at the application-level, i.e. whether differences in star ratings were explained primarily by features or the individual application.

## 3 Results

### 3.1 Topic structure

The semi-automated topic modeling using NLP resulted in eight final topics: The app (49% occurrence); Immersiveness and/or VR-related sensory topics (42%); Technically related topics (38%); Relaxation and/or purpose with application 34%); Criticism (19%); Games and gamification (17%); Functionality, variety and options in app (16%); and Price (13%). The Criticism topic collected predominantly negative sentiments, although the technically related topic also had a high prevalence of negative sentiment. The remaining topics expressed either predominantly positive, neutral or both these sentiments, for an overall sentiment score of 66% positive, 18% neutral and 16% negative (sentiments detected in 82% of reviews). Both the immersion-related topic and relaxation-related topics included terms suggesting that the applications were successful in inducing relaxation and showed predominantly positive sentiments. Terms included in the Criticism topic covered both technical (“overheating”), design (“creepy”) and usage (“bored”) issues. A topic covering terms relating to functionality and variety of options in the application had a low occurrence (16%) yet showed predominantly positive sentiment, suggesting that such features were appreciated in the applications with these options. The games and gamification topic too had a low occurrence, yet was 69% positive. See Table 2 for full results.

**Table 2:** Final topic structure.

Topic	Terms^a^	Occurrence %	Negative sentiment %	Positive sentiment %	Neutral sentiment %
The app	App, experience, vr, new, apps, experiences, overall, application, experienced, general, all in all, applications, mobile app, thorough, experiance, journeys, general experience, comprehensive, thourough, vr-experience, interactive	48.87	10.97	69.43	19.58
Immersiveness and/or VR-related sensory topics	Music, graphics, environments, environment, developers, look, realistic, scenes, scenery, looks, sound, video, immersive, looking, scene, 3d, visuals, 360, devs, developer, imagery, details, style, 2d, photo, atmosphere, animation, 180, surroundings, landscapes, animations, sunset, distraction, photorealistic, scenic, design, noise, quiet, photo-realistic, cartoony, art style, sceneries, beaches, designs, monoscopic, 360 degrees, silent, songs, animated	41.76	10.76	66.49	22.74
Technically related topics	Work, update, download, oculus go, tried, install, try, oculus, trying, change, working, controller, works, downloaded, updates, device, downloading, uninstall, worked, installed, changes, changing, installing, updated, tries, reinstalled, updating, reinstalling, vr headset, installation, reinstall, oculus rift, attempts, changed, installs, oculus go store, gear vr, uninstalling, uninstalled, controllers, disable, gamepad	38.21	19.92	37.95	42.12
Relaxation and/or purpose with application	Relaxing, meditation, relax, relaxation, quite, calm, calming, meditate, relaxed, peaceful, guided meditation, soothing, focus, meditations, meditative, chill, helped me, meditating, tranquil, breathing exercises, calm down, helped, contemplative, mindfulness, de-stress, tranquility, calmer, calmed, focus/relax, meditation room, relaxation time, quieter, helping, quiteness, tranquilize, roam freely, loose time, great help	34.3	7.18	69.13	23.67
Criticism	Please, fix, bad, fixed, disappointed, stress, disappointing, overheating, annoying, opposite, overheats, avoid, terrible, bored, awful, unrealistic, solve, not enough, pointless, useless, mediocre, pls, solved, overheat, frustrated, underwhelming, dissapointed, creepy, stressed out, resolve, solution, resolved, wierd, confused, inconsistent, intrigued, overheated, lacking, crappy, peeved, sketchy, lacks, disconcerting, skeptical, messed, anxious, fixing, insufficient	19.36	48.31	24.71	26.96
Games and gamification	Game, fishing, games, play, fish, playing, gameplay, sailing, multiplayer, puzzle game, game play	16.96	6.83	69.23	23.93
Functionality, variety and options in app	Options, locations, option, different, close, area, variety, areas, near, range, selection, selections, varied, wide selection, assortment, different types, diverse, different styles	16.31	11.55	54.22	34.22
Price	Worth, bought, money, pay, buy, price, buying, cost, cheap, priced	12.54	16.18	35.83	47.97

a Includes misspelt words that occurred in the corpus.

### 3.2 Quantitative findings

**Fig. 3:**
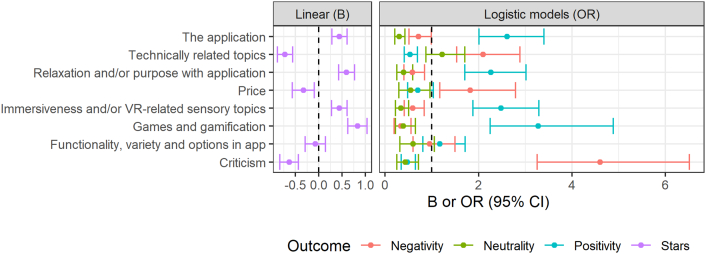
Multiple regression models predicting star rating and sentiment from topics.

**Fig. 4:**
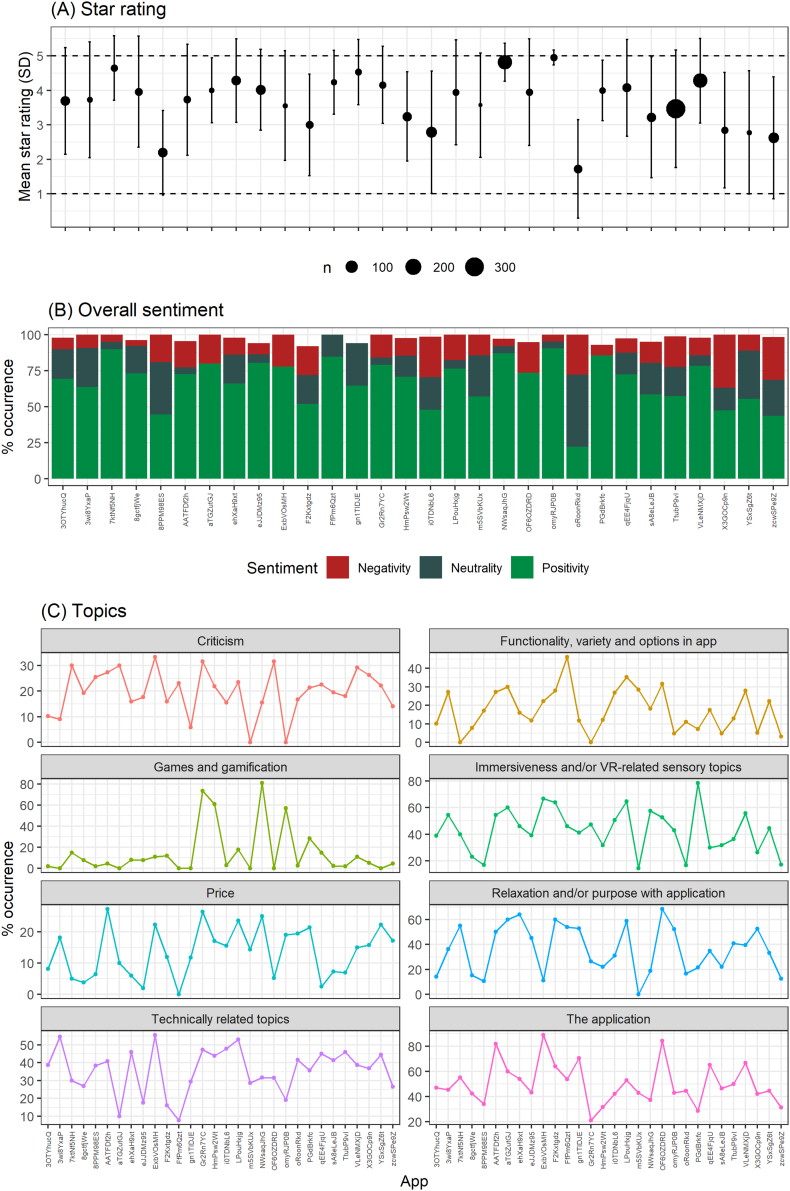
Distribution of star ratings, sentiments and topics across applications.

Results from multiple logistic regression models predicting each overall sentiment using all topics were largely congruent with within-topic sentiments. Congruently, four topics were positively associated with star rating, three were negatively associated, and one had no significant association. See Fig. 3 for parameter estimates and odds ratios (including 95% confidence intervals) from the multiple regression models. Star ratings significantly differed across applications (Welch's F_29, 200_ = 26.9, *p* < .001), as did occurrence of positive, negative and neutral overall sentiments (respectively; all p < .001, χ_29_ > 69.89). All topic occurrences except Criticism (*p* = .173, χ_29_ = 36.0) also differed significantly across applications (all *p* < .003, χ_29_ > 54.19). See Fig. 4 for full results. In the ordinary linear regression model predicting star ratings from all four categorization variables, passive applications (B = -0.74, SE = 0.18, p < .001) and those with unnatural environments (B = -0.52, SE = 0.16, *p* = .001) received lower ratings, while type and price were not significant associated with star rating (*p* = .21 and *p* = .57, respectively). However, when rerunning this regression model as a random-intercept mixed model (clustering by application), no predictors remained significant, with random intercepts accounting for 29% of the variance.

## 4 Discussion

VR is a growing consumer technology, and relaxation applications – of which there are now many available at digital marketplaces – have quickly become one of the most popular of non-entertainment genres. While there is mounting evidence from laboratory studies that VR relaxation is efficacious (Anderson et al., 2017; Annerstedt et al., 2013; Hedblom et al., 2019; Serrano et al., 2016; Valtchanov et al., 2010) and appreciated by users (Seabrook et al., 2020), there has been no research on how these applications are used in everyday life, how they are viewed by users under real-world conditions, or how different application features are associated with user reception. The current study addresses these key research gaps by mining online user reviews of such VR relaxation applications, extracting topics through semi-automated NLP, and examining statistical associations between topics, sentiments and star ratings. In sum, we find that users have an overall positive view of these applications, describing them as successful in inducing immersion and relaxation, and having many appreciated features such as gamification elements and options to tailor the relaxation experience. As hypothesized, there was however great variation across applications in terms of star ratings, review sentiments and topics, suggesting that the quality of each individual application is more important than specific aspects of the application (e.g. type of virtual relaxation environment offered).

Immersion- and relaxation-related topics were associated with positive sentiments being expressed and greater star ratings, suggesting that these applications are indeed successful in inducing relaxation, replicating findings from laboratory studies. The occurrence of these topics did however differ greatly between applications, as did star ratings, revealing substantial differences in perceived quality across applications and indirectly suggesting difference in effectiveness. This mirrors past research on consumer smartphone applications, which have been found to vary considerably in both use of evidence-based therapeutic components (Shen et al., 2015), direct empirical support (Linardon et al., 2019) and use of scientific language to support these claims (Larsen et al., 2019). The vast differences in perceived quality of VR relaxation applications may stem in part from the low threshold of application development for this particular type of application, with a minimally viable product requiring only 360° videos of nature environments, soothing music and a minimal user interface. This has spurred a commercial interest in the genre, made even more attractive by the promise of prescription business models since relaxation offers only temporary relief for what is often a chronic issue; this in contrast with other clinical applications of VR such as exposure therapy for phobias (Donker et al., 2019; Freeman et al., 2018; Lindner et al., 2020b; Miloff et al., 2019) that are intended to be used during a specific timeframe, after which symptoms are expected to have decreased more or less permanently (i.e. no further need for the application). While the observed variation in perceived VR application quality is not surprising given findings on smartphone applications, market mechanisms like open reviews and star ratings may hopefully pivot users towards high-quality applications. Moreover, exploring how VR users choose mental health applications is arguably an important future research question for the field that should be explored using both account tracking studies and qualitative research.

The fact that user reception varied considerably between applications and that application-level clustering of star ratings was considerable, makes it difficult to draw general conclusions on what types of features that are viewed positively, suggesting instead that overall application quality and user experience is more important than any specific feature. Findings from this first explorative study nonetheless suggest some implications for the design of VR relaxation applications, which should be explored further in future studies. The games and gamification topic, for example, had an overall low occurrence rate but showed occurrence spikes (>50%) in reviews of four applications that also showed high average star ratings and high occurrence of positive sentiments. Congruently, on a review-level, this topic was associated with greater positive sentiment occurrence and higher star ratings, even in a multiple regression model that accounted for occurrence of other topics (application-level clustering should however be considered). Although these analyses do not directly contrast gamified vs non-gamified applications on the basis of an independent classification – this aspect was not included in the classification scheme due to difficulties in thresholding – our results indicate that some applications make greater use of gamification elements (and is mentioned by reviewers), and that these applications are better received by users.

A detailed, comparative case series description of gamification elements included in specific applications is beyond the scope of the current study, yet we observed that gamification elements included common ones like accomplishment badges, promotion of usage statistics (e.g. number of completed sessions), and leadership boards (Koivisto and Hamari, 2019), as well as building the relaxation experience around a soothing activity (e.g. fishing) that requires no physical activity (detrimental to relaxation) and can be easily gamified. It should be noted that the specific terms covered by the topic focused on the latter and in the regression model that did not cluster variance in star ratings on application, passive applications (i.e. not built around a gamified, relaxing activity) were rated lower. Further, there was great variation in the occurrence and implementation of these elements in the reviewed apps, making it inappropriate to draw conclusions on which particular gamification elements that were appreciated. Although gamification elements are increasingly used in digital mental health applications, evidence of efficacy of specific elements is scarce (Johnson et al., 2016). However, previous qualitative research on both VR interventions for mental (Lindner et al., 2020c) and physical health (Faric et al., 2019), as well as non-VR interventions for mental health (Cheek et al., 2015), suggest that such features are indeed appreciated. Our results replicate these findings using a complementary method, supporting the hypothesis that it is advantageous to include them; in lieu of direct experimental support, appreciated features can be hypothesized to increase efficacy by promoting engagement and adherence. Well-marketed, high-quality consumer VR applications are today likely to find tens of thousands of users in a short timeframe (Lindner et al., 2019), in principle allowing randomized A/B testing and/or interrupted time series analyses of individual (gamification) features on an unprecedented and unrivaled scale. This would offer power to disentangle even small effects of specific gamification elements. Carrying out such research in industry-academia collaborations would be of great value not only in providing users with iteratively more efficacious VR relaxation applications, but would also be of great value to the gamification field in general. Further in-depth qualitative research on how users perceive specific gamification elements in VR mental health applications is also needed.

Similarly, the question whether tailoring capabilities in application result in greater efficacy and/or engagement lacks a definitive answer in the extant literature. Findings from the current study suggest that although variety and options showed a positive within-topic sentiment, it was not associated with any sentiment or star rating. This may be an effect of the overall low occurrence of this topic, an overall low tendency of users to refer to such features in their review when features were available and used (misclassification bias), and/or differences across applications in availability, quality of implementation, within-application promotion, and extent of actual use. Although no clear design implications regarding this particular type of feature can be drawn from findings of the current study, the extant literature does suggest that extended functionality in the form of tailoring capabilities could be advantageous. Preliminary laboratory research on VR relaxation using 360° video environments suggests that different nature scenes have different relaxing effects (Wang et al., 2019) and greater effects than urban environments (Hedblom et al., 2019). Further, VR relaxation effects correlate somewhat with preference for different nature environments (Gao et al., 2019), although it remains unknown how well such effects generalize across populations and graphical modalities. In addition, there is indirect evidence from outside the VR field emphasizing the importance of offering a tailored experience, e.g. research showing that sedative and stimulative music were found to be equally relaxing if the music was individually preferred and chosen, but not when it was non-preferred (Jiang et al., 2013). Given the great individual variability in what kind of music and visual environment one finds soothing, offering users the option to tailor their experience offers a convenient way around this issue, although the value of this approach has yet to be tested empirically.

As in previous qualitative research on VR relaxation (Seabrook et al., 2020) and gamified VR exposure therapy (Lindner et al., 2020c), several negative experiences emerged, both technical and usage-related. Strikingly, this topic was the only one that did not differ significantly in occurrence across applications, and overall topic occurrence was almost 20%. Although this study did not include a comparison on criticism occurrence with other types of VR applications, our findings show that a relatively large minority of reviewing users experience issues with what is often relatively simple applications software-wise. Inspecting included terms reveals that users report issues with the installation process, overheating, that updates rendered the application broken, or took issue with the visual presentation (“creepy”, “unrealistic”) or gameplay (“confused”). Users also report negative (opposite of intended) effects (Rozental et al., 2014), indicated by terms like “stress”, “stressed out”, “anxious” and “annoying”. Aside from obvious glitches and bugs (which can largely be avoided through careful programming), it is possible that some of these criticisms may stem from personal preferences, e.g. whether a perfectly photorealistic but passive 360° video nature environment is preferred over an interactive, cartoon-looking environment. Again, this risk may be avoidable by offering the user a variety of environments, potentially reducing the risk of few recurring users (Lindner et al., 2019), as also observed with smartphone applications for mental health (Baumel et al., 2019). In-depth qualitative research, along with experimental research that actively induces different bugs and glitches (Liebold et al., 2017), will be required to examine exactly how different criticism impact relaxation efficacy and subsequent application use.

Of interest, we found a low occurrence of terms associated with cybersickness (Kennedy et al., 1993), a state resembling motion sickness believed to be caused primarily by a sensory discrepancy between the visual and vestibular/proprioception systems (Rebenitsch and Owen, 2016) although visual presentation, locomotion, duration and even user characteristics also play a direct or indirect role (Saredakis et al., 2020). This low occurrence is not surprising given recent improvements in hardware capabilities (e.g. lower latency, higher resolution) and specifically the type of VR devices surveyed in the current study (3DOF, not allowing any physical locomotion). Further, relaxation experiences seldom feature content known to increase the risk of cybersickness, such as moving the first-person view or including rapid, large movements of stimuli in the environment (Chang et al., 2020), since this would be detrimental to relaxation. The material and topic modeling in the current study suggests (through absence) that VR cybersickness is not a prominent issues in modern, consumer-targeted relaxation applications, at least with 3DOF devices. To what degree this is moderated by specific content types does however remain to be investigated: due to the low overall occurrence of cybersickness terms, great variation in content between applications and likely also in user usage patterns (in applications that features customization options), we are unable to estimate whether certain types of relaxation experiences (e.g. slowly moving through a virtual landscape vs sitting still) have a higher degree of reports of cybersickness. Such specific hypotheses are better tested in randomized experiments with detailed used behavior logging and/or by contrasting reviews of specific application that are similar with regards to other content.

Although the price topic showed predominantly neutral or even positive within-topic sentiments, it was negatively associated with star ratings and increased odds of negative overall review sentiment when included in multiple regression models that also adjusted for the occurrence of other topics. These regression models did not take application into account, and in addition to having an overall low occurrence rate of 12.54%, occurrence rates differed significantly between applications, appearing in 0–25% of reviews depending on application. In addition, free applications did not differ in star ratings from non-free applications, although this result may be confounded by changes in pricing model since release, of which no record was available. Together, these findings suggest that users found some applications overpriced and others worth the price, emphasizing the need for a balance between application quality and cost which should be carefully considered by developers, possibly adjusted regularly to account for growing consumer expectations. The fact that users found some free applications appealing, and are willing to pay for applications of higher quality, is promising for the dissemination of such applications and the potential of a significant public health impact by offering a low-threshold, efficacious way of dealing with stress and other common mental health problems, as VR continues to grow as a consumer technology (Lindner, 2020).

### 4.1 Strengths and limitations

Strengths of the current study include a large sample of reviews of thirty applications for popular, low-cost VR devices, ensuring low sampling bias and allowing us to generalize findings beyond evaluation of individual applications. The semi-automated NLP analysis complements traditional qualitative approaches and enables synergistic quantitative analyses. Several limitations nonetheless apply. First, while the NLP model makes the entire processing pipeline reproducible (and in principle applicable to other corpuses of text), it was still manually guided. Second, for reasons listed above, the current study was limited to reviews of applications available for the Oculus Go and Gear VR devices, categorized and explicitly described as a relaxation application on the official digital marketplace, at a specific point in time. Other applications may since have been added or removed from the specific store category and/or the store itself, before or after time of data collection. To what degree the findings of the current study extend to other VR applications with relaxation components and to applications for other VR devices, and to what degree the findings remain applicable over time as consumers are likely to become more demanding, remains to be investigated.

Third, although the thresholds for writing and publishing a review of an Oculus Go application are low, reviewing users nevertheless present a sample of the larger population who used each application, the representativeness of which is unknown. Any sampling bias can however be expected to be much smaller than if explicitly recruiting for a study on user experiences. Complementary research using objective measures of application usage linked to individual accounts would provide a fully unbiased, quantitated view of user experience, albeit a narrow one. It would theoretically be possible to link online reviews with objective application usage data through account usernames, yet this would require special logging procedures, user consent, and developers making such linked data available.

Fourth, in this first explorative study, we opted for a relatively low level of detail in the application content classification scheme, prioritizing robustness over specificity. This was deemed even more appropriate given that many applications had seen content changes since time of release (a detailed record of which was not available) and since the presence of customization features means that mere availability of specific content does not equate to user experiences thereof. For this reason, we refrained, for example, from attempting to separate user experiences of mindfulness exercises versus somatic relaxation (both offered e.g. by the Calm Place application), which may have different psychological effects (Jain et al., 2007). Arguably, it would be more appropriate to examine such contrasts within the context of a randomized trial, by using time-series analysis of reviews of a specific application before and after feature addition, or similar.

Finally, due to the inherent complexities of human language, along with the diversity of use and occurrence of human errors, sentiment analysis is unlikely to ever yield perfect results: accuracy rates of text-level analyses (the level of analysis in the current study) are typically in the range of 70–90% (Karlgren et al., 2012). In the current study, sentiments from the topic model showed associations with star ratings in the expected direction, suggesting overall satisfactory validity even in the presence of occasional incorrect classifications of individual reviews.

### 4.2 Conclusions

In this first, explorative study, we found that users have an overall positive view of VR relaxation applications, which appear to be largely successful in providing relaxation. The many applications differed considerably in average star rating and also in the degree to which reviews covered different topics. A relatively large minority of participants expressed criticism covering both technical issues and content presentation. Gamification elements appear to be appreciated features, and free applications were not rated higher, suggesting that users are willing to pay for higher quality applications.

## CRediT authorship contribution statement

Simon Fagernäs^:^ Methodology, Formal analysis, Writing - Review & Editing

William Hamilton: Software, Investigation, Writing - Review & Editing

Nicolas Espinoza: Methodology, Resources, Writing - Review & Editing

Alexander Miloff: Investigation, Data Curation, Writing - Review & Editing

Per Carlbring: Conceptualization, Writing - Review & Editing

Philip Lindner: Conceptualization, Methodology, Data curation, Formal analysis, Investigation, Methodology, Project administration, Supervision, Visualization, Writing - Original Draft

## Declaration of competing interest

Author WH is a founder and chief technology officer of Mimerse, which developed one of the applications reviews of which were analyzed in the current study. Author PL has consulted for Mimerse but holds no financial stake in the company. Applications names were blinded at an early stage of preprocessing, and neither WH nor PL were involved in developing the topic structure. Author NE is a founder of and business developer at Gavagai, the tool used for NLP.
